# Structure of LP2179, the first representative of Pfam family PF08866, suggests a new fold with a role in amino-acid metabolism

**DOI:** 10.1107/S1744309109023689

**Published:** 2009-10-27

**Authors:** Constantina Bakolitsa, Abhinav Kumar, Dennis Carlton, Mitchell D. Miller, S. Sri Krishna, Polat Abdubek, Tamara Astakhova, Herbert L. Axelrod, Hsiu-Ju Chiu, Thomas Clayton, Marc C. Deller, Lian Duan, Marc-André Elsliger, Julie Feuerhelm, Slawomir K. Grzechnik, Joanna C. Grant, Gye Won Han, Lukasz Jaroszewski, Kevin K. Jin, Heath E. Klock, Mark W. Knuth, Piotr Kozbial, David Marciano, Daniel McMullan, Andrew T. Morse, Edward Nigoghossian, Linda Okach, Silvya Oommachen, Jessica Paulsen, Ron Reyes, Christopher L. Rife, Henry J. Tien, Christina V. Trout, Henry van den Bedem, Dana Weekes, Qingping Xu, Keith O. Hodgson, John Wooley, Ashley M. Deacon, Adam Godzik, Scott A. Lesley, Ian A. Wilson

**Affiliations:** a Joint Center for Structural Genomics, http://www.jcsg.org, USA; b Program on Bioinformatics and Systems Biology, Burnham Institute for Medical Research, La Jolla, CA, USA; c Stanford Synchrotron Radiation Lightsource, SLAC National Accelerator Laboratory, Menlo Park, CA, USA; d Department of Molecular Biology, The Scripps Research Institute, La Jolla, CA, USA; e Center for Research in Biological Systems, University of California, San Diego, La Jolla, CA, USA; f Protein Sciences Department, Genomics Institute of the Novartis Research Foundation, San Diego, CA, USA; g Photon Science, SLAC National Accelerator Laboratory, Menlo Park, CA, USA

**Keywords:** structural genomics, DUFs, *S*-adenosylmethionine decarboxylase, amino-acid metabolism, probiotics

## Abstract

The structure of LP2179, a member of the PF08866 (DUF1831) family, suggests a novel α+β fold comprising two β-sheets packed against a single helix. A remote structural similarity to two other uncharacterized protein families specific to the *Bacillus* genus (PF08868 and PF08968), as well as to prokaryotic *S*-adenosylmethionine decarboxylases, is consistent with a role in amino-acid metabolism. Genomic neighborhood analysis of LP2179 supports this functional assignment, which might also then be extended to PF08868 and PF08968.

## Abstract 2

The first structural representative of the PF08866 (DUF1831) protein family reveals a potential new α+β fold and indicates a possible involvement in amino-acid metabolism.

## 1. Introduction

The Pfam database (Finn *et al.*, 2008 ▶) contains over 2000 domains of unknown function (DUFs), which are protein families for which the biological function is unknown and cannot be deduced by homology. Currently, DUFs are the best source for the discovery of new folds (Jaroszewski *et al.*, submitted), followed by large families with no structural representatives. DUF structures provide the first step towards establishing functional hypotheses and extending our understanding of the protein universe. In an effort to sample and understand the diversity of protein-fold and structure space, targets were selected from Pfam protein family PF08866 (DUF1831). Here, we report the crystal structure of LP2179, the first structural representative of this family, which was determined using the semiauto­mated high-throughput pipeline of the Joint Center for Structural Genomics (JCSG; Lesley *et al.*, 2002 ▶) as part of the NIGMS Protein Structure Initiative (PSI; http://www.nigms.nih.gov/Initiatives/PSI/). The *LP2179* gene of *Lactobacillus plantarum*, a lactic acid-producing bacterium found in human saliva and intestinal flora, encodes a protein with a molecular weight of 12.6 kDa (residues 1–113) and a calculated isoelectric point of 8.9. LP2179 appears to adopt a novel fold with remote similarities to proteins with a TBP-like fold (TATA-binding protein), including *S*-adenosyl-l-methionine decarboxylase (EC 4.1.1.50), an enzyme implicated in the urea cycle and the catabolism of methionine and amino groups. Analysis of the genomic neighborhood of DUF1831 homologs reveals the systematic presence of other enzymes implicated in amino-acid and amino-group metabolism, suggesting a similar role for other members of the DUF1831 family and for two other functionally uncharacterized families that show partial structural similarity to LP2179.

## 2. Materials and methods

### 2.1. Protein production and crystallization

Clones were generated using the Polymerase Incomplete Primer Extension (PIPE) cloning method (Klock *et al.*, 2008 ▶). The gene encoding LP2179 (GenBank NP_785678, gi:28378786, Swiss-Prot Q88V95) was amplified by polymerase chain reaction (PCR) from *L. plantarum* WCFS1 NCIMB8826 genomic DNA using *PfuTurbo* DNA polymerase (Stratagene) and I-PIPE (Insert) primers (forward primer, 5′-ctgtacttccagggcATGGCATACACAACAACGGTTAAAC-3′; reverse primer, 5′-­aattaagtcgcgttaGTCCGTCGTGAGGATATCCCGTTC-3′; target sequence in upper case) that included sequences for the predicted 5′ and 3′ ends. The expression vector pSpeedET, which encodes an amino-terminal tobacco etch virus (TEV) protease-cleavable expression and purification tag (MGSDKIHHHHHH­ENLYFQ/G), was PCR-amplified with V-PIPE (Vector) primers (forward primer, 5′-taacgcgacttaattaactcgtttaa­acggtctccagc-3′; reverse primer, 5′-gccctggaagtacaggttttcgtgatgatgat­gatgatg-3′). V-PIPE and I-­PIPE PCR products were mixed to anneal the amplified DNA fragments together. *Escherichia coli* GeneHogs (Invitrogen) com­petent cells were transformed with the V-PIPE/I-PIPE mixture and dispensed onto selective LB–agar plates. The cloning junctions were confirmed by DNA sequencing. Expression was performed in a selenomethionine-containing medium. At the end of fermentation, lysozyme was added to the culture to a final concentration of 250 µg ml^−1^ and the cells were harvested and frozen. After one freeze–thaw cycle, the cells were sonicated in lysis buffer [50 m*M* HEPES pH 8.0, 50 m*M* NaCl, 10 m*M* imidazole, 1 m*M* tris(2-­carboxyethyl)phosphine–HCl (TCEP)] and the lysate was clarified by centrifugation at 32 500*g* for 30 min. The soluble fraction was passed over nickel-chelating resin (GE Healthcare) pre-equilibrated with lysis buffer, the resin was washed with wash buffer [50 m*M* HEPES pH 8.0, 300 m*M* NaCl, 40 m*M* imidazole, 10%(*v*/*v*) glycerol, 1 m*M* TCEP] and the protein was eluted with elution buffer [20 m*M* HEPES pH 8.0, 300 m*M* imidazole, 10%(*v*/*v*) glycerol, 1 m*M* TCEP]. The eluate was buffer-exchanged with TEV buffer (20 m*M* HEPES pH 8.0, 200 m*M* NaCl, 40 m*M* imidazole, 1 m*M* TCEP) using a PD-10 column (GE Healthcare) and incubated with 1 mg of TEV protease per 15 mg of eluted protein. The protease-treated eluate was run over nickel-chelating resin (GE Healthcare) pre-equilibrated with HEPES crystallization buffer (20 m*M* HEPES pH 8.0, 200 m*M* NaCl, 40 m*M* imidazole, 1 m*M* TCEP) and the resin was washed with the same buffer. The flowthrough and wash fractions were combined and concentrated to 10 mg ml^−1^ by centrifugal ultrafiltration (Millipore) for crystallization trials. LP2179 was crystallized by mixing 200 nl protein solution with 200 nl crystallization solution in a sitting-drop format over a 50 µl reservoir volume using the nanodroplet vapor-diffusion method (Santarsiero *et al.*, 2002 ▶) with standard JCSG crystallization protocols (Lesley *et al.*, 2002 ▶). Crystals from two different crystallization conditions were used for data collection and structure determination. The crystallization reagent yielding a cube-like crystal (0.1 × 0.1 × 0.1 mm) used for MAD phasing consisted of 20.0%(*w*/*v*) PEG 6000 and 0.1 *M* Bicine pH 9.0 as the precipitant. A long rod-like crystal (0.3 × 0.1 × 0.1 mm) used for refinement was obtained using 0.2 *M* NaCl, 20.0%(*w*/*v*) PEG 8000 and 0.1 *M* CAPS pH 10.5. Crystallization was carried out at 277 K for both conditions. Glycerol was added to both crystals as a cryoprotectant to a final concentration of 15%(*v*/*v*). Initial screening for diffraction was carried out using the Stanford Automated Mounting system (SAM; Cohen *et al.*, 2002 ▶) at the Stanford Synchrotron Radiation Lightsource (SSRL, Menlo Park, California, USA). Both sets of diffraction data were indexed in the orthorhombic space group *P*2_1_2_1_2_1_ (Table 1 ▶). The oligomeric state of LP2179 was determined using a 0.8 × 30 cm^2^ Shodex Protein KW-803 column (Thomson Instruments) pre-calibrated with gel-filtration standards (Bio-Rad).

### 2.2. Data collection, structure solution and refinement

Multiple-wavelength anomalous diffraction (MAD) data were collected at the Advanced Photon Source (APS, Argonne, Illinois, USA) on beamline 23-ID-D at wavelengths corresponding to the high-energy remote (λ_2_), inflection (λ_3_) and peak (λ_4_) of a selenium MAD experiment. Higher resolution data from a different crystal were collected at the Advanced Light Source (ALS, Berkeley, California, USA) on beamline 8.2.2. The data sets were collected at 100 K using a MAR Mosaic 300 detector (APS) and an ADSC Quantum-315 CCD detector (ALS). The MAD data were integrated and reduced using *XDS* and then scaled with the program *XSCALE* (Kabsch, 1993 ▶). The higher resolution (λ_1_) data were integrated and reduced using *MOSFLM* (Leslie, 1992 ▶) and then scaled with the program *SCALA* (Collaborative Computational Project, Number 4, 1994 ▶). Phasing of the MAD data was performed with *SOLVE* (Terwilliger & Berendzen, 1999 ▶; four selenium sites per asymmetric unit, mean FOM = 0.52) and automated model building was performed with *ARP*/*wARP* (Cohen *et al.*, 2004 ▶). The resulting model was used for model completion and refinement against the higher resolution (λ_1_) data with *Coot* (Emsley & Cowtan, 2004 ▶) and *REFMAC* 5.2 (Murshudov *et al.*, 1999 ▶). Data reduction and refinement statistics are summarized in Table 1 ▶.

### 2.3. Validation and deposition

**Figure 1:**
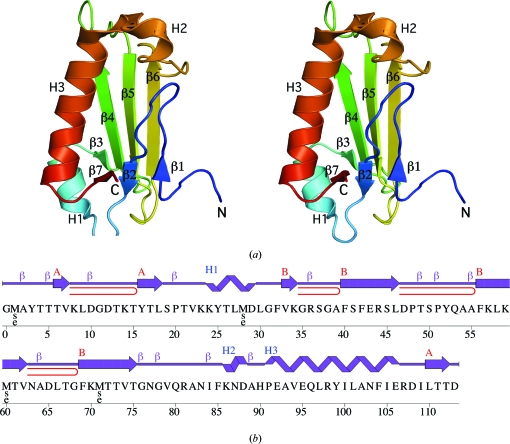
Crystal structure of LP2179 from *L. plantarum*. (*a*) Stereo ribbon diagram of the LP2179 monomer color-coded from the N-terminus (blue) to the C-terminus (red). Helices H1–H3 and β-strands (β1–β7) are indicated. (*b*) Diagram showing the secondary-structure elements of LP2179 superimposed on its primary sequence. The labeling of secondary-structure elements is in accord with *PDBsum* (http://www.ebi.ac.uk/pdbsum), where α-helices are sequentially labeled (H1, H2, H3 *etc*), β-strands are labeled (A, B, C *etc*.) according to the β-sheets to which they are assigned, β-turns and γ-turns are designated by Greek letters (β, γ) and β-hairpins by red loops. For LP2179, the α-­helices (H1–H3), β-sheets (A, B) and β-turns (β) are indicated. Selenomethionine residues used for phasing are labeled MSE.

Analysis of the stereochemical quality of the model was accomplished using *AutoDepInputTool* (Yang *et al.*, 2004 ▶), *MolProbity* (Davis *et al.*, 2004 ▶), *SFCHECK* 4.0 (Collaborative Computational Project, Number 4, 1994 ▶) and *WHATIF* 5.0 (Vriend, 1990 ▶). Protein quaternary-structure analysis used the *PISA* server (Krissinel & Henrick, 2007 ▶). Fig. 1 ▶(*c*) was adapted from an analysis using *PDBsum* (Laskowski *et al.*, 2005 ▶) and all other figures were prepared with *PyMOL* (DeLano Scientific). Atomic coordinates and experimental structure factors for LP2179 at 1.20 Å resolution have been deposited in the PDB under accession code 2iay.

## 3. Results and discussion

### 3.1. Overall structure

The crystal structure of LP2179 (Fig. 1 ▶ *a*) was initially determined to 1.33 Å resolution using the multiple-wavelength anomalous dispersion (MAD) method and was further refined to 1.20 Å resolution using data collected from a different crystal. Data-collection, model and refinement statistics are summarized in Table 1 ▶. The final model includes 114 residues (*i.e.* the residual Gly0 from the expression tag followed by residues 1–113 of LP2179), one glycerol molecule, one chloride ion and 195 water molecules in the asymmetric unit. The side chains of Lys8, Lys59 and Lys86 were not modeled owing to poor electron density. The Matthews coefficient (*V*_M_; Matthews, 1968 ▶) is 2.0 Å^3^ Da^−1^ and the estimated solvent content is 37.2%. The Ramachandran plot produced by *MolProbity* (Davis *et al.*, 2004 ▶) shows that 98.2% and 100% of the residues are in favored and in favored and additionally allowed regions, respectively.

LP2179 forms a single domain composed of two antiparallel β-­sheets packed against a long C-terminal helix H3 (Fig. 1 ▶). A second helix, H1, links strand β2 from the first β-sheet (order 127), which is assembled from the two N-terminal and the C-terminal β-strands, to the second β-sheet (order 3456) and packs parallel to H3. *Pre-SCOP* classifies LP2179 as a novel fold termed LP2179-like (http://www.mrc-lmb.cam.ac.uk/agm/pre-scop/999384.html). Analysis of the crystallographic packing of LP2179 using the *PISA* server (Krissinel & Henrick, 2007 ▶) and analytical size-exclusion chromatography in combination with static light scattering indicate that a monomer is the likely quaternary form.

### 3.2. Comparison with other structures

**Figure 2:**
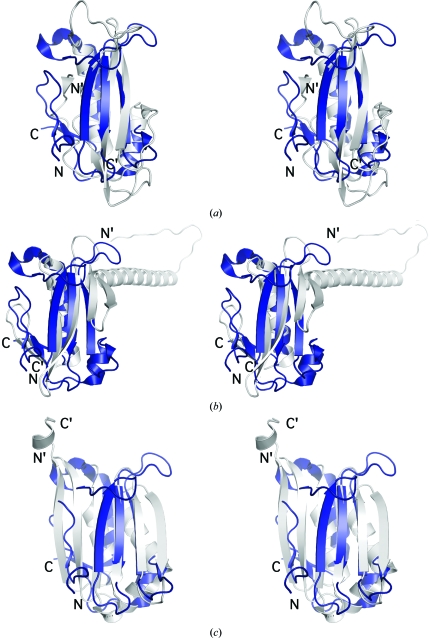
LP2179 exhibits structural similarity to members of the YugN-like family, DUF1185 and *S*-adenosylmethionine decarboxylases. Stereoviews of the structural superposition of LP2179 (PDB code 2iay, in blue) with (in gray) (*a*) a YugN-like homolog from *B. clausii* (PDB code 2pww), (*b*) a DUF1885 homolog from *B. stearothermophilus* (PDB code 1t6a) and (*c*) *S*-adenosylmethionine decarboxylase proenzyme (TM0655) from *Thermotoga maritima* (PDB code 1vr7). N- and C-termini are indicated for LP2179 and are indicated with primes (N′, C′) for other structures.

A search with *FATCAT* (Ye & Godzik, 2004 ▶) revealed a remote structural similarity of LP2179 to members of the YugN-like family (PF08868), which are characterized by a TBP-like fold (http://www.mrc-lmb.cam.ac.uk/agm/pre-scop/55944. html). Superposition of LP2179 onto ABC2387 (PDB code 2pww; U. A. Ramagopal, J. Freeman, C. Lau, R. Toro, K. Bain, L. Rodgers, J. M. Sauder, S. K. Burley & S. C. Almo, unpublished work), a YugN-like homolog from *Bacillus clausii*, clearly reveals that both proteins share the same fold and topology over all of the helices and strands β3–β5 from the second β-sheet (strands β3–β6; Fig. 2 ▶ *a*). The structural similarity involves a main-chain r.m.s.d. of 2.5 Å over 81 residues, although the sequence identity is only 7%. Similar values are obtained for GK1089, another YugN homolog from *Geobacillus kaustophilus* (PDB code 2r5x), with an r.m.s.d. of 2.9 Å and 10% sequence identity over 87 aligned residues. Both YugN-like homologs show an interruption in the regular hydrogen-bonding pattern of strand β6 in the β-sheet, resulting in two shorter, collinear strands that hydrogen bond separately to β5. However, as the TBP-like fold is characterized by a β-α-β_4_-α topology, the main topological difference between the two families involves the first β-sheet in LP2179, which is replaced in YugN-like homologs by a β-strand that forms part of the single β-­sheet (Fig. 2 ▶ *a*). The H2 helix, which is absent in both YugN-like and DUF1885 homologs, might constitute an additional difference, but owing to its short size (one helical turn) and its involvement in crystal contacts (Asp88–Arg107′ and Phe85–Arg107′) it might not represent a biologically relevant conformation of this region in solution.

A search with *FFAS* (Jaroszewski *et al.*, 2005 ▶) showed no significant sequence similarity of LP2179 to any protein family other than PF08866. However, significant sequence similarity (*FFAS* score −11 with 20% sequence identity) was observed between ABC2387 and RBSTP2229, a member of the protein family PF08968 (DUF1885) from *B. stearothermophilus*. Like the YugN-like homologs, RBSTP2229 also exhibits a TBP-like fold. A structural superposition of ABC2387 (PDB code 2pww) with RBSTP2229 (PDB code 1t6a) shows a backbone r.m.s.d. of 2.8 Å over 57 residues. Over the same residue range, LP2179 has a backbone r.m.s.d. of 3.3 Å with RBSTP2229 (Fig. 2 ▶ *b*). However, the length and orientation of helix H1 in RBSTP2229 (pointing outwards from the structure instead of packing against the central β-sheet) differs substantially from that observed in ABC2387 and LP2179, while the subsequent β-strand is positioned differently with respect to helix H3 in all three structures (Figs. 2 ▶ *a* and 2 ▶ *b*). Among these TBP-like variants, LP2179 is unique in that the N- and C-terminal β-strands are combined to form an additional β-sheet that is situated between the central β-sheet and helix H3. However, both YugN-like and DUF1885 homologs display shorter variants of this secondary-structure element in the same region (YugN-like homologs contain a single β-strand; DUF1185 forms a C-terminal hairpin), raising the possibility that this region might represent a locus in this family for structural and possibly functional drift (Krishna & Grishin, 2005 ▶).

Structural similarities of LP2179 to prokaryotic *S*-adenosyl­methionine decarboxylases (AdoMetDCs; EC 4.1.1.50) were also observed. Superposition of LP2179 onto the AdoMetDC from *Thermotoga maritima* results in a backbone r.m.s.d. of 3.3 Å over 82 residues with 3% sequence identity (Fig. 2 ▶ *c*). Similar values (an r.m.s.d of 3.3 Å over 67 residues with 3% sequence identity) were obtained for the AdoMetDC from *Aquifex aeolicus* (PDB code 2iii). As with the YugN-like homologs, prokaryotic AdoMetDCs share a similar fold and topology as LP2179 that includes the main β-sheet (β3–β6) and helices (H1–H3) in addition to the C-terminal β-strand (β7) of LP2179. The main differences involve the arrangement of the N- and C-terminal β-strands in prokaryotic AdoMetDCs that hydrogen bond to form a single six-stranded antiparallel β-sheet, as opposed to the two separate sheets in LP2179, and a C-terminal helix that is absent in LP2179 (Fig. 2 ▶ *c*).

Structural comparison between these four Pfam families reveals the conservation of a core β-α-β_4_-α (TBP-like) fold with β-strand additions at the N- or C-terminus or both. In LP2179, a strand is added at both the N- and C-termini, while YugN-like homologs contain an extra β-strand at the N-terminus (topology β_2_-α-β_4_-α) and PF08968 homologs contain an additional β-strand at the C-terminus that follows a circular permutation of the core fold (topology α-β_4_-α-β_2_). AdoMetDCs contain an additional β-strand at the C-terminus that hydrogen bonds to the N-terminal strand to form an antiparallel six-stranded β-sheet (topology β-α-β_4_-α-β).

It is widely accepted that protein structure is more conserved than amino-acid sequence, suggesting that structural relationships between proteins might provide information that is not available from sequence alone (see review by Kolodny *et al.*, 2006 ▶). Both the PF08866 (DUF1831) and PF08868 (YugN-like) protein families are currently functionally uncharacterized. AdoMetDC is a pyruvoyl-dependent amino-acid decarboxylase that is involved in methionine metabolism and is essential for polyamine biosynthesis (Pegg *et al.*, 1998 ▶). The structure of prokaryotic AdoMetDC proenzyme (Toms *et al.*, 2004 ▶) reveals that despite the lack of any detectable sequence similarity between the eukaryotic and prokaryotic forms of the enzyme (13% sequence identity), the two structures can be superimposed with an r.m.s.d. of 2.0 Å for 156 backbone residues. The catalytic site residues are also conserved (Toms *et al.*, 2004 ▶).

**Figure 3:**
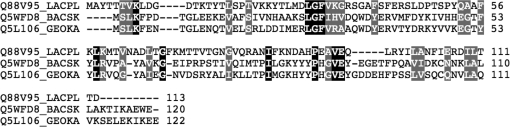
Sequence alignment of LP2179 and members of the YugN-like family. UniProt abbreviations are as follows: Q88V95_LACPL, gene locus lp_2179 from *L. plantarum*; Q5WFD8_BACSK, gene locus ABC2387 from *B. clausii*; Q5L106_GEOKA, gene locus GK1089 from *G. kaustophilus*. Residues are shaded by identity (black) and similarity (gray).

The AdoMetDC proenzyme undergoes an autocatalytic intra­molecular self-cleavage reaction that generates a pyruvoyl group in a loop between two β-strands (β3 and β4 in Fig. 2 ▶ *c*). Although the catalytic residues (Ser and Glu) of the AdoMetDC proenzyme are not conserved in LP2179 and YugN-like or Pfam08968 homologs, sequence alignment reveals the conservation of charged and aromatic residue clusters between LP2179 and YugN-like homologs (Fig. 3 ▶). In the respective structures, these clusters occur along the first two strands and intervening loop of the central β-sheet (β3 and β4 in Figs. 1 ▶ *a* and 2 ▶ *c*) surrounding the AdoMetDC catalytic site and may serve a similar functional role.

### 3.3. Genomic neighborhood analysis

The genomic neighborhood (http://string.embl.de) of LP2179 shows a high degree of confidence in a predicted functional association with cysteine desulfurase (LP2180, score 0.81) and methylthioadenosine nucleosidase (LP2181, score 0.64). Cysteine desulfurase (EC 2.8.1.7) catalyzes the production of alanine from cysteine, while methylthioadenosine nucleosidase (EC 3.2.2.16) also participates in the metabolism of amino groups. These two enzymes are found in the genomic context or neighborhood of every member of the DUF1831 family, supporting a role for DUF1831 in amino-acid metabolism.

In Gram-positive bacteria, such as the *Bacillus* genus, amino-acid metabolism is directly coupled to several other metabolic pathways, including trans-sulfuration, polyamine synthesis and recycling, the activated methyl cycle and quorum sensing (Lebeer *et al.*, 2007 ▶). As previously indicated, AdoMetDC is a central regulator of these pathways. Modified amino acids, such as homocysteine, or their catabolic products, such as polyamines, can serve both pathogenic and probiotic roles. In pathogenic bacteria, polyamines and homocysteine are involved in biofilm formation (Shah & Swiatlo, 2008 ▶; Abraham, 2006 ▶), with polyamines also being implicated in bacterio­cin production and protection from acid and oxidative stress (Shah & Swiatlo, 2008 ▶). The probiotic role of lactobacilli has been well documented (Ryan *et al.*, 2008 ▶); their antimicrobial activity results from the production of bacteriocins and antifungal peptides (De Vuyst & Leroy, 2007 ▶). Further work will be required to determine whether the fold similarities observed between the *Bacillus* protein families described in this paper translate into similarities in function and whether this function might involve a probiotic role.

The availability of more DUF1831 sequences and structures might shed light on the evolutionary history of this intriguing protein family. The information presented here, in combination with further biochemical and biophysical studies, should yield valuable in­sights into the functional role of LP2179. Models for LP2179 homologs can be accessed at http://www1.jcsg.org/cgi-bin/models/get_mor.pl?key=2iayA.

Additional information about the protein described in this study is available from TOPSAN (Krishna *et al.*, 2010 ▶) http://www.topsan.org/explore?PDBid=2iay.

## 4. Conclusions

The first structural representative of the DUF1831 family reveals a potential new fold with remote similarities to TBP-like structures. This similarity, in combination with genomic context analysis, leads us to propose an involvement in amino-acid metabolism that might also be extended to two other families of unknown function.

## Supplementary Material

PDB reference: LP2179 from *L. plantarum*, 2iay, r2iaysf

## Figures and Tables

**Table 1:** Summary of crystal parameters, data-collection and refinement statistics for LP2179 (PDB code 2iay) Values in parentheses are for the highest resolution shell.

	λ_1_ Se	λ_2_ MADSe	λ_3_ MADSe	λ_4_ MADSe
Data collection				
Space group	*P*2_1_2_1_2_1_	*P*2_1_2_1_2_1_		
Unit-cell parameters (Å)	*a* = 36.29, *b* = 47.90, *c* = 58.01	*a* = 36.41, *b* = 47.99, *c* = 57.83		
Wavelength (Å)	0.9798	0.9493	0.9794	0.9793
Resolution range (Å)	28.9–1.2 (1.23–1.20)	29.0–1.3 (1.36–1.33)	29.0–1.4 (1.41–1.37)	29.0–1.4 (1.41–1.37)
No. of observations	181436	69961	63756	63463
No. of unique reflections	29115	21577	19702	19718
Completeness (%)	90.4 (50.7)	90.4 (49.2)	90.2 (46.3)	90.3 (47.2)
Mean *I*/σ(*I*)	16.4 (2.1)	13.3 (2.0)	12.9 (2.0)	12.9 (1.7)
*R*_merge_ on *I*† (%)	6.9 (20.4)	6.2 (38.8)	6.2 (37.9)	7.2 (46.7)
*R*_meas_ on *I*‡ (%)	7.3 (26.7)	7.3 (52.4)	7.3 (51.1)	8.5 (63.1)
Model and refinement statistics				
Resolution range (Å)	28.9–1.2			
No. of reflections (total)	29080§			
No. of reflections (test)	1488			
Completeness (%)	90.1			
Data set used in refinement	λ_1_ Se			
Cutoff criterion	|*F*| > 0			
*R*_cryst_¶	0.120			
*R*_free_¶	0.147			
Stereochemical parameters				
Restraints (r.m.s.d. observed)				
Bond angles (°)	1.65			
Bond lengths (Å)	0.016			
Average isotropic *B* value (Å^2^)	8.86			
ESU†† based on *R*_free_ (Å)	0.038			
Protein residues/atoms	114/944			
Water molecules/other solvent	195/2			

† *R*_merge_ = 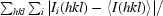 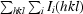.

‡ *R*_meas_ = 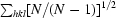 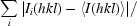 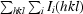 (Diederichs & Karplus, 1997 ▶).

§ Typically, the number of unique reflections used in refinement was slightly less that the total number that were integrated and scaled. Reflections were excluded owing to systematic absences, negative intensities and rounding errors in the resolution limits and unit-cell parameters.

¶ *R*_cryst_ = 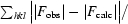 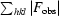, where *F*_calc_ and *F*_obs_ are the calculated and observed structure-factor amplitudes, respectively. *R*_free_ is the same as *R*_cryst_ but for 5.1% of the total reflections chosen at random and omitted from refinement

†† Estimated overall coordinate error (Collaborative Computational Project, Number 4, 1994 ▶; Tickle *et al.*, 1998 ▶).
